# Specific gut bacterial responses to natural diets of tropical birds

**DOI:** 10.1038/s41598-022-04808-9

**Published:** 2022-01-13

**Authors:** Kasun H. Bodawatta, Irena Klečková, Jan Klečka, Kateřina Pužejová, Bonny Koane, Michael Poulsen, Knud A. Jønsson, Katerina Sam

**Affiliations:** 1 Natural History Museum of Denmark, University of Copenhagen, Copenhagen, Denmark; 2 Biology Centre of Czech Academy of Sciences, Institute of Entomology, Branisovska 31, 37005 Ceske Budejovice, Czech Republic; 3 Faculty of Science, University of South Bohemia, Branisovska 1760, 37005 Ceske Budejovice, Czech Republic; 4 New Guinea Binatang Research Centre, Madang, Papua New Guinea; 5 Section for Ecology and Evolution, Department of Biology, University of Copenhagen, Copenhagen, Denmark

**Keywords:** Microbial ecology, Tropical ecology, Ecology, Microbiology, Bacteria, Microbial communities

## Abstract

The composition of gut bacterial communities is strongly influenced by the host diet in many animal taxa. For birds, the effect of diet on the microbiomes has been documented through diet manipulation studies. However, for wild birds, most studies have drawn on literature-based information to decipher the dietary effects, thereby, overlooking individual variation in dietary intake. Here we examine how naturally consumed diets influence the composition of the crop and cloacal microbiomes of twenty-one tropical bird species, using visual and metabarcoding-based identification of consumed diets and bacterial 16S rRNA microbiome sequencing. We show that diet intakes vary markedly between individuals of the same species and that literature-based dietary guilds grossly underestimate intraspecific diet variability. Furthermore, despite an effect of literature-based dietary guild assignment of host taxa, the composition of natural diets does not align with crop and cloacal microbiome similarity. However, host-taxon specific gut bacterial lineages are positively correlated with specific diet items, indicating that certain microbes associate with different diet components in specific avian hosts. Consequently, microbiome composition is not congruent with the overall consumed diet composition of species, but specific components of a consumed diet lead to host-specific effects on gut bacterial taxa.

## Introduction

The composition of gut microbial communities of animals is driven by a multitude of intrinsic (i.e., host genetics, immune system)^1,2^ and extrinsic (i.e., diet, environment)^3–6^ factors. In many animal taxa, the establishment of the initial microbiome is facilitated by the inoculation of microbial consortia from parents (parental transmission)^7,8^. However, the colonisation and persistence of these microbes can be influenced by both the host immune system and gut physiology^2,9,10^. Throughout host life, ecological and environmental factors (environmental filtering) such as diet, habitat, and social interactions further affect composition and stability (i.e., individual variation and turnover rates) of bacterial communities^5,6,11–15^. Thus, to understand the evolution and the long-term associations between hosts and their gut microbes, we need to disentangle the relative importance of these factors.

Of the ecological and environmental factors, diet has been shown to have a strong influence on shaping the gut microbiomes of many wild animals^3,4,6,16^. This is particularly true in birds^4,10,11,13^. However, most studies on gut microbiomes in wild birds have relied on the literature to assign bird taxa to particular dietary guilds^10,17–19^, with a few exceptions where stable isotope ratios of nitrogen and carbon have been used to characterize the nutrient composition of natural diets^20,21^. The utilisation of literature-based dietary guilds ignores individual and seasonal natural variation in diet intake^22^, which may be important for the gut microbiome composition^11,21^. Furthermore, overall dietary guild assignments to e.g., frugivore and insectivore tend to strongly associate with host taxonomy (closely related species tend to belong to similar feeding guilds)^23^, hindering the ability to determine the realized separate effects of host taxonomy and diet. Thus, to elucidate the realized effect of diet on structuring wild bird gut microbiomes, it is necessary to examine naturally consumed diet items of individuals, which is currently lacking in wild avian gut microbiome research.

**Figure 1:**
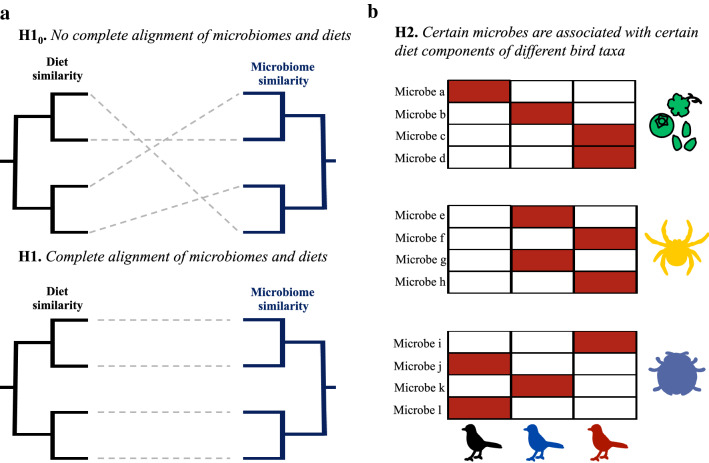
A conceptual overview of the investigated diet-related hypotheses. (**a**) Hypothesis 1: gut microbiome similarity is strongly associated with consumed diet similarity across all bird species irrespective of host phylogeny. (**b**) Hypothesis 2: host species-specific gut microbes are associated with certain diet (three different diets depicted) items, where different bird species (three different species depicted) harbour different microbial taxa that are associated with the same diet items. Red boxes represent significant correlations between diet components and gut microbes, while white boxes represent no associations.

In an attempt to reduce this knowledge gap, we examine how natural diets (as opposed to expected diets based on the literature) of wild birds influence the crop (the food storing pouch) and the cloacal bacterial communities of tropical forest birds in Papua New Guinea. Through the collection of regurgitated crop samples, we characterised specific consumed diet contents and crop microbiomes. These microbiomes could represent both incoming microbes of the ingested diet and the microbes that are already present in the crop. Utilising cloacal swabs, we then investigated the cloacal microbiomes of the same individuals. Diet identification in the crop was conducted using two commonly utilised approaches: DNA metabarcoding and visual identification, while crop and cloacal microbiomes were characterized through sequencing the v4 region of the bacterial 16S rRNA gene. First, we tested two alternative hypotheses related to the effect of diets on crop and cloacal microbiomes (Fig. 1). If consumed diet similarity is a strong determinant of wild avian gut microbiomes, we expected individuals consuming compositionally similar diets to harbour similar crop and cloacal microbiomes, irrespective of host taxonomy (Fig. 1a). However, if host taxon is the main driver, and diet only secondarily influences community structure^10^, we did not expect microbiome similarity to align with diet similarity across host taxa. Instead, we expected significant correlations between specific microbes and diet items across hosts (Fig. 1b). Secondly, we compared the crop and cloacal microbiomes of the same individuals, testing the assumption that the avian stomach acts as a barrier for passage of microbes from the foregut to the hindgut^24^, expecting that microbial communities in the crop and the cloaca would be compositionally different. Anatomical gut modifications associated with powered flight has led to gut microbial restrictions in birds (e.g., increased individual variation and less stability of gut microbiomes in smaller birds)^9,10^, and we therefore further predicted that smaller birds with shorter digestive tracts^10^ would have more shared bacterial sequences in the two regions.

## Results

### Natural diets of tropical birds vary within species

**Figure 2:**
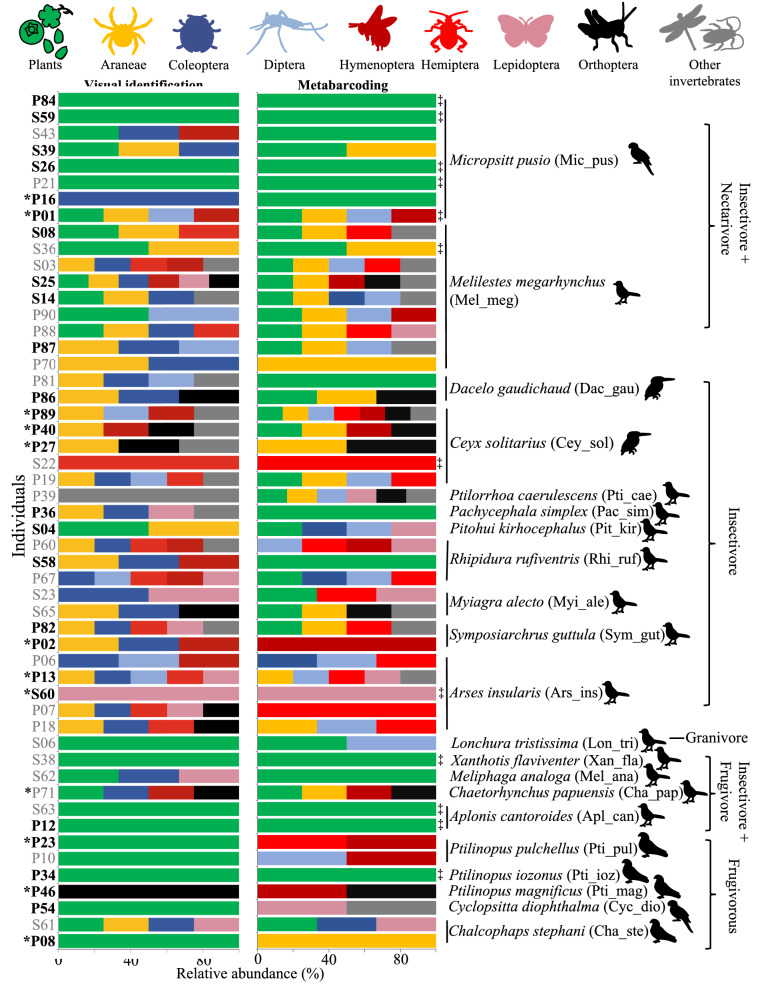
Natural diets of wild birds vary between individuals of the same species and the results of the two identification methods of dietary components (visual identification and metabarcoding). Relative abundances based on the presence/absence of data of different dietary components are indicated in colours. Only invertebrates are separated into taxonomic orders as visual identification is unable to identify plant orders. Individuals depicted with asterisks had both crop microbiome and diet samples (dataset 1), while black font represents individuals with both cloacal microbiomes and diet samples (dataset 2). Individuals are clustered according to the species (each species is given a six-letter code name) and their literature-based dietary guilds. The order of the species is indicated with illustrations (Columbiformes﻿–Pigeons, Coraciiformes﻿–Kingfishers, Passeriformes﻿–Passerines and Psittaciformes﻿–Parrots), while ‡ represents diet samples with a complete consensus between the two identification methods.

We collected 62 regurgitated samples (using the tartar emetic method ^22^) from multiple tropical bird species representing four bird orders (Columbiformes–Pigeons, Coraciiformes–Kingfishers, Psittaciformes–Parrots, and Passeriformes–Passerines). First, we characterized diet components visually and then through metabarcoding of 52 of these samples using universal primers targeting invertebrates (Cytochrome c oxidase subunit I: COI gene) and plants (Internal transcribed spacer 2: ITS2 gene) (Table S1 and Fig. 2). Through visual identification, we identified plant material in 26 samples. The most common visually identified invertebrate orders were Araneae (spiders—27 samples), and Coleoptera (beetles—27 samples) (Table S2). Metabarcoding sequences were analysed using the OBITools software^25^. Overall, we found 47 plant operational taxonomic units (OTUs—97% sequence similarity threshold) and 180 invertebrate OTUs (Table S3). Plant items were dominated by the orders Rosales (27.7% OTUs), Fabales (8.5% OTUs), and Sapindales (8.5% OTUs). Except for four OTUs, all plants were identified to the genus level. Of the invertebrate OTUs, 54 belonged to feather mites (known feather symbionts), endoparasites, and rotifers (likely due to accidental consumption along with drinking water), and these OTUs were removed from further analyses, leaving 126 potential dietary invertebrate OTUs. Invertebrate samples were dominated by the classes Insecta (67.5% OTUs) and Arachnida (28.6% OTUs). At the order-level, dietary items were mainly represented by Araneae (spiders—28.6% OTUs), Hemiptera (true bugs—15.9% OTUs), Diptera (flies—14.3% OTUs), and Lepidoptera (moths and butterflies—10.3% OTUs). However, 77% of the invertebrate OTUs could not be identified to genus level, highlighting the limited research on genotyping invertebrate communities in Papua New Guinea.

Diet item identification differed markedly between visual and metabarcoding methods (Fig. 2, Tables S2 and S3). The diet components of individuals also varied notably within species (Figs. 2 and S1). Only diets of 12 out of 52 individuals were fully congruent between the two methods (Fig. 2). Of these 12 samples, eight had only plant material. Identification of invertebrate orders also differed between the two methods (Fig. 2, Table 1). Both methods identified the arthropod orders Hemiptera, Diptera, Orthoptera (crickets and locusts), and Araneae in the same samples (Fig. 2 and Table 1), while metabarcoding detected lower proportions of Coleoptera than the visual identification (Table 1).

**Table 1:** Comparison between diet items identified in the regurgitated samples from the two approaches (visual identification and metabarcoding).

Diet component	Visual ID	Metabarcoding	# of samples with consensus between the two methods	% of samples with visual ID of diet components matching the metabarcoding results (%)
Plants	26	37	22	84.6
Araneae	27	23	19	70.4
Coleoptera	27	5	4	14.8
Diptera	9	16	8	88.9
Hymenoptera	12	11	7	58.3
Hemiptera	11	15	11	100
Lepidoptera	9	9	4	44.4
Orthoptera	9	9	7	77.8

Identification of plants is limited to the domain level in the visual method. Invertebrate identification is done at order level to enable comparison between the two methods.

### Comparison of microbiomes and consumed diet items

For subsequent comparisons of diets and microbiomes, we utilised individual datasets from both visual identification (diet components identified at the order level) and metabarcoding (both OTU and order level), and a combination (order level) of both approaches (for details see “Methods” section on identifying prey items). Due to differences between the diet identification methods, a combination of the results was used to circumscribe the full diversity of consumed diets and to account for inherent biases associated with the two methods (i.e., the inability to identify plant material and smaller body parts of invertebrates visually, and extraction and sequencing biases associated with metabarcoding). We separated the microbiome dataset into three datasets due to sequencing limitations: dataset 1 included 12 birds with successfully sequenced crop microbiomes and diets identified using both methods, dataset 2 included 27 birds with successfully sequenced cloacal microbiomes and diets, and dataset 3 included 17 birds for which we obtained successfully sequenced crop and cloacal microbiomes (Table S1). Prior to subsequent analyses, each microbiome dataset was rarefied to even sequencing depths using the sample with the lowest number of sequences^26^ (Fig. S2).

### Crop microbiome similarity did not align with the consumed diet similarity (dataset 1)

Out of the collected crop samples (N = 62), samples from only 19 individuals were successfully sequenced for their microbiomes. Of these individuals, we acquired diet samples for 12 individuals. Bacterial 16S rRNA MiSeq sequences were analysed using the DADA2 pipeline^27^ within QIIME2^28^. There were 351,867 bacterial sequences (mean ± SD: 29,322 ± 33,009) in the crop microbiomes prior to rarefaction (Table S4). After rarefaction, bacterial sequences were identified to 615 amplicon sequence variants (ASVs—100% sequence similarity). Crop microbiomes were dominated by Proteobacteria (53.6%), Actinobacteria (18.9%), and Firmicutes (17.9%). Alpha diversities of individual microbiomes were calculated using the diversity function in the microbiome package^29^ and they did not differ significantly between host orders [Chao1 richness: Kruskal Wallis (KW) χ^2^ = 4.559, df = 3, *p* = 0.2271; Shannon’s diversity index: χ^2^ = 2.853, df = 3, *p* = 0.4149], or literature-based dietary guilds (Chao1 richness: KW χ^2^ = 4.317, df = 2, *p* = 0.1155; Shannon’s diversity index: KW χ^2^ = 2.852, df = 2, *p* = 0.2403) (Fig. S3).

The compositional differences of crop microbiomes were investigated with the adonis2 function in the vegan package^30^ using permutational multivariate analyses of variance tests (PERMANOVA). These analyses revealed that the bird host order did not influence the crop microbiome composition (PERMANOVA_10,000 permutations_: Bray–Curtis: F = 1.251, R^2^ = 0.0993, *p* = 0.1911; Jaccard: F = 1.154, R^2^ = 0.0962, *p* = 0.2191) (Fig. S1). The effect of feeding guild was masked by host order as they are strongly correlated in this dataset. Furthermore, the lack of an effect of host taxa on crop microbiomes may be a result of the small sample sizes.

We further investigated whether alpha diversity of the crop microbiomes was influenced by the diet item diversity of individuals. The Chao1 richness estimates of the microbiomes and the richness of the consumed diet items (number of different diet items based on the combined results) of individuals were not significantly correlated (Table S5), suggesting that the diet richness does not impact crop microbiome richness. However, Shannon’s diversity index of crop microbiomes and diet diversity were marginally significantly negatively associated (Table S5). This suggests that despite the lack of an association between diet and microbiome richness, crop microbiome evenness could be influenced by diet diversity.

We then explored the association between the crop microbiome composition and the consumed diets, investigating correlations between Bray–Curtis and Jaccard dissimilarities of microbiomes, and Jaccard dissimilarity of diets using Mantel tests in the vegan package^30^. The compositional similarity of the diets based on any of the methods (visual, metabarcoding—both OTU and order-level separately, and combined) did not correlate significantly with crop microbiome compositions (Table 2 and Fig. S4). We observed similar non-significant associations between diets and microbiomes when investigating host orders separately (Table S6). This suggests that overall crop microbiomes of individuals are not completely modelled by the composition of the consumed diets.

**Table 2:** Results of Mantel tests between the crop (dataset 1) and the cloacal (dataset 2) microbiome similarities (measured with both Bray–Curtis and Jaccard distances) and the consumed diet similarities (measured with Jaccard distances).

Dataset	Diet identification	Microbiome distance matrix	Mantel r	*p*
Dataset 1 (Crop microbiomes and diets)	Visual	Bray–Curtis	0.1017	0.2165
		Jaccard	0.0861	0.2438
	Metabarcoding (order level)	Bray–Curtis	0.0373	0.3736
		Jaccard	0.0186	0.4231
	Metabarcoding (OTU level)	Bray–Curtis	− 0.0844	0.7113
		Jaccard	− 0.0839	0.7064
	Combined	Bray–Curtis	0.0368	0.3958
		Jaccard	0.0077	0.4542
Dataset 2 (Cloacal microbiomes and diets)	Visual	Bray–Curtis	0.0586	0.1531
		Jaccard	0.0449	0.1955
	Metabarcoding (order level)	Bray–Curtis	0.0379	0.2176
		Jaccard	0.0425	0.1947
	Metabarcoding (OTU level)	Bray–Curtis	0.1285	0.0511
		Jaccard	0.1159	0.0609
	Combined	Bray–Curtis	− 0.0284	0.6392
		Jaccard	− 0.0176	0.5745

Separate tests were conducted for visual, metabarcoding (both at diet OTU and order level) and the combined identification of diets.

### Host-taxon specific cloacal microbes are associated with different diet items (dataset 2)

We obtained 27 individuals from 15 bird species with successfully sequenced cloacal microbiomes and diet samples (based on both metabarcoding and visual identification). Prior to rarefying, we acquired 818,272 bacterial sequences from the cloacal swab samples (mean ± SD: 30,306 ± 20,903) (Table S7). After rarefaction, bacterial sequences were assigned to 1,324 ASVs that belonged to Actinobacteria (35.9%), Proteobacteria (32.6%), Firmicutes (21.2%) and Tenericutes (5.0%). Cloacal microbiome alpha diversity did not differ significantly between different bird orders (Chao1 richness: KW χ^2^ = 2.624, df = 3, *p* = 0.4532; Shannon’s diversity: χ^2^ = 6.595, df = 3, *p* = 0.0861) or literature-based dietary guilds (Chao1 richness: KW χ^2^ = 1.128, df = 3, *p* = 0.7703; Shannon’s diversity: KW χ^2^ = 1.673, df = 3, *p* = 0.6429) (Fig. S5).

**Figure 3:**
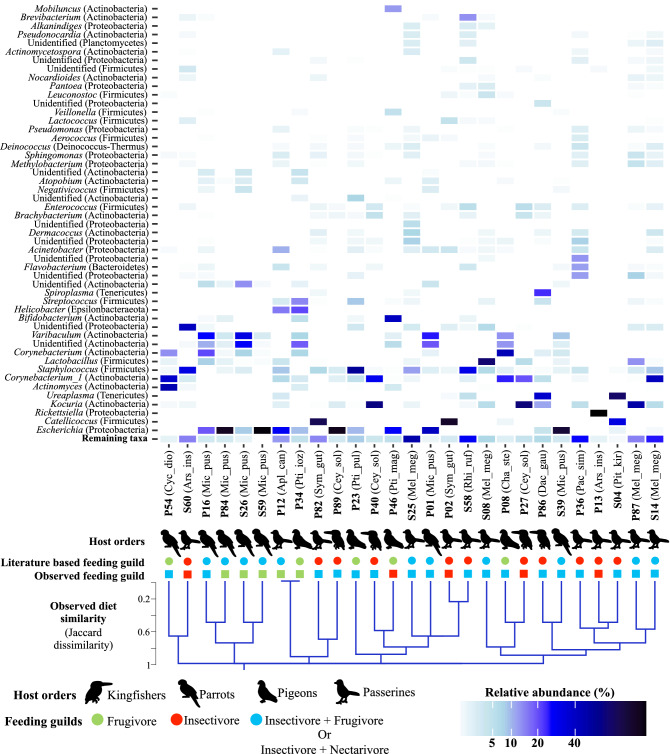
Overall cloacal microbiomes were not influenced by the observed diet similarity of individuals. The heatmap depicts the relative abundance of the 50 most abundant bacterial genera in the cloacal microbiomes. The dendrogram represents the consumed diet similarity (combined dataset) between individuals based on Jaccard distances. Literature-based dietary guilds and the observed feeding guilds of the individuals are indicated below the bird order icons. Insectivore + Frugivore and Insectivore + Nectarivore dietary guilds have been combined since none of the diet identification methods was able to identify nectar. The species code of each individual is given near the host ID number (code names from Fig. 2).

However, cloacal microbiome beta diversity was significantly influenced by host bird order (PERMANOVA_10,000 permutations_: Bray–Curtis: F = 2.159, R^2^ = 0.2055, *p* < 0.0001; Jaccard: F = 1.749, R^2^ = 0.1775, *p* < 0.0001) and literature-based dietary guilds (PERMANOVA_10,000 permutations_: Bray–Curtis: F = 1.529, R^2^ = 0.1456, *p* = 0.0008; Jaccard: F = 1.341, R^2^ = 0.1361, *p* = 0.0023) (Fig. S1). The variation explained by dietary guilds was slightly secondary to the variation explained by the host taxon. We did not observe significant associations between alpha diversity of diet and cloacal microbiomes (Table S5) nor in compositional similarity of diets and cloacal microbiomes (Table 2 and Fig. 3). Similar to crop microbiomes, the compositional similarity of diets and cloacal microbiomes were not significantly associated when bird orders were analysed separately (Table S6). This suggests that cloacal microbiome composition does not align with the overall consumed diet similarities of hosts.

**Figure 4:**
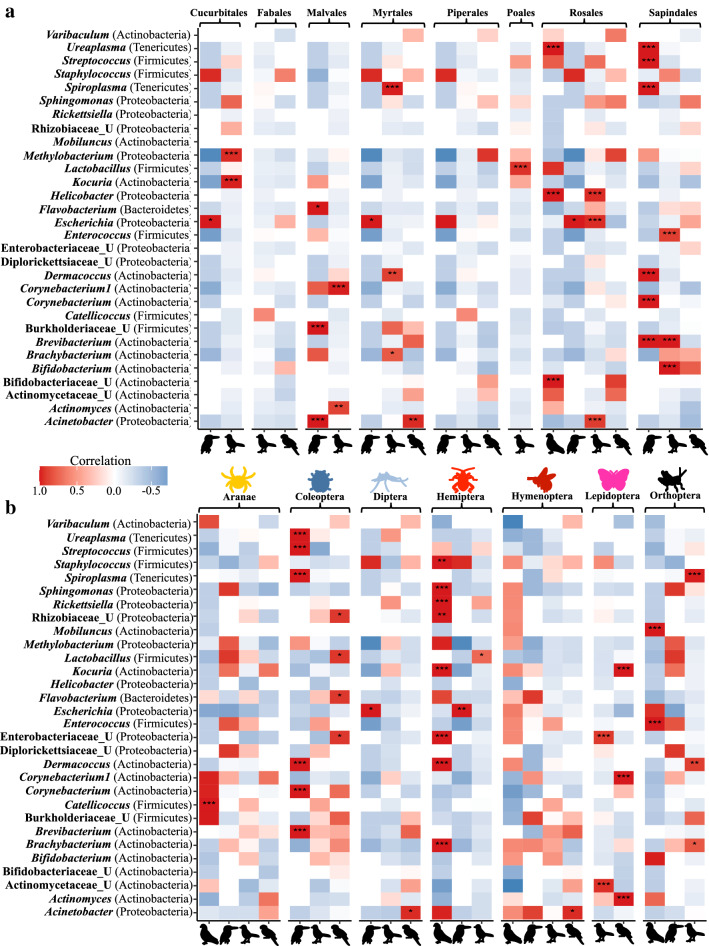
Many host-taxon-specific bacterial genera are significantly positively correlated with the relative abundance of certain dietary components. Pearson’s correlations between the 30 most abundant cloacal bacterial genera and the proportion of different orders of plants (**a**) and invertebrates (**b**) in individual diets. These analyses were conducted only on the diet identification based on metabarcoding, as visual identification did not identify plants into lower-level taxonomic classifications. Significant correlations are indicated with asterisks (*p* < 0.05*, *p* < 0.001**, and *p* < 0.0001***).

To explore whether specific gut bacterial symbionts of different host taxa are associated with different dietary items (Fig. 1b), we tested for correlations between the 30 most abundant bacterial genera and the proportion of order-level diet items in each individual using the taxa.env.correlation function in the microbiomeSeq package^31^. These analyses revealed that certain bacterial genera were positively correlated with certain dietary items (Fig. 4 and Table S8) and that the taxonomy of bacterial symbionts associated with the same diet item differed between host orders, suggesting that host-taxon specific microbes are affected by the same dietary items in different bird taxa. For example, the relative abundance of the plant order Rosales was significantly correlated with the bacterial genera *Ureaplasma*, and *Helicobacter* in pigeons, while Rosales was significantly associated with *Helicobacter*, *Escherichia* and *Acinetobacter* in passerine birds. These results indicate that the overall effect of diet on cloacal microbiomes results from a combination of associations between certain microbes and specific dietary items in different avian hosts.

### A large portion of bacterial sequences are shared between the crop and the cloaca (dataset 3)

**Figure 5:**
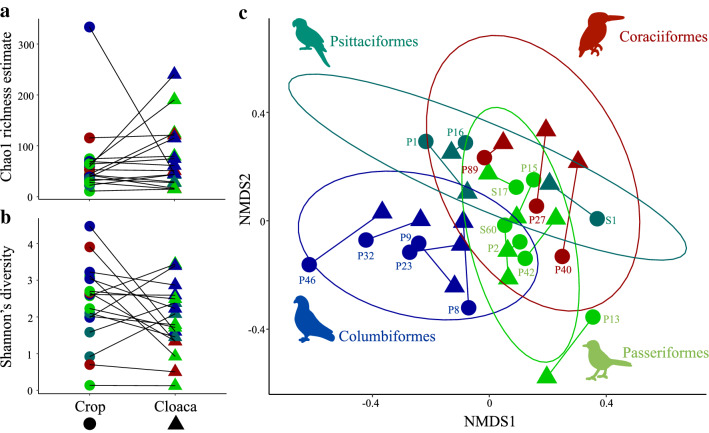
Alpha diversities did not differ between crop and cloacal microbiomes, but beta diversities demonstrated a host order-level effect. Chao1 richness estimate (**a**) and Shannon’s diversity index (**b**) of microbiomes in the two regions of the digestive tract. Microbiomes of the same bird individual are connected with a line. (**c**) The NMDS plot (stress = 0.2243) represents the microbial community similarity (measured with Jaccard dissimilarity index) of the cloacal and the crop microbiomes. Individual IDs are given near the crop samples and points are coloured according to the host order. Ellipses represent the 95% confidence intervals.

**Figure 6:**
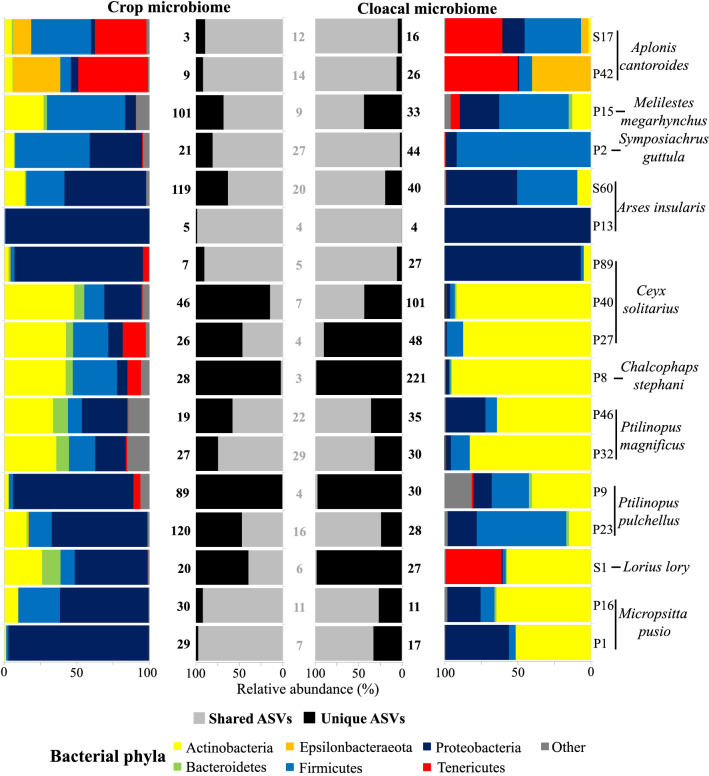
Crop and cloacal microbiomes share a large proportion of abundant bacterial ASVs. The far left and far right panels show the relative abundance of bacterial phyla in the crop and the cloacal microbiomes of the same individual. The two panels in the middle show the proportion of bacterial sequences (relative abundance) belonging to the shared and unique ASVs in the two regions. The number of unique ASVs are shown in black and shared ASVs are shown in grey.

For 17 bird individuals, we successfully acquired both crop and cloacal microbiomes. Overall, prior to rarefaction, we acquired 571,488 (mean ± SD: 33,617 ± 17,188) bacterial sequences from cloacal swab samples and 562,557 sequences from crop samples (mean ± SD: 33,091 ± 35,586). After rarefaction, sequences aligned to 1,176 bacterial ASVs (Table S9). The microbiome alpha diversity did not differ significantly between the two regions (Chao1 richness: KW χ^2^ = 0.3633, df = 1, *p* = 0.5466; Shannon’s diversity index: KW χ^2^ = 1.759, df = 1, *p* = 0.1848) (Fig. 5a,b). Overall, the phylum Proteobacteria (43.9%) dominated the crop microbiomes, followed by Firmicutes (20%) and Actinobacteria (19%). In the cloaca, microbiomes were dominated by Actinobacteria (40.2%), followed by Proteobacteria (25.1%) and Firmicutes (21.7%) (Fig. 6). The relative abundance of the bacterial phyla in both regions of the digestive tract differed markedly between bird species but we observed comparable microbial compositions within species (Fig. 6). Overall, bacterial community compositions did not differ significantly between the two regions of the gut (PERMANOVA_10,000 permutations_: Bray–Curtis: F = 0.9188, R^2^ = 0.0279, *p* = 0.5985; Jaccard: F = 0.8995, R^2^ = 0.0273, *p* = 0.6825) (Fig. 5c), while both the crop (PERMANOVA_10,000 permutations_: Bray–Curtis: F = 1.661, R^2^ = 0.2771, *p* = 0.0026; Jaccard: F = 1.393, R^2^ = 0.2432, *p* = 0.0051) and the cloacal (PERMANOVA_10,000 permutations_: Bray–Curtis: F = 1.721, R^2^ = 0.2841, *p* < 0.0001; Jaccard: F = 1.521, R^2^ = 0.2597, *p* = 0.0006) microbiomes were significantly affected by host order (Fig. 5c). The similarity between the crop and the cloacal microbiomes within individuals indicates that microbiomes are likely to be influenced by the same factors, e.g., host taxon.

Only a small number of ASVs (richness) was shared between the crop and the cloacal microbiomes (mean ± SD: 17.1% ± 12.2%). However, they accounted for a large proportion of the total number of bacterial sequences (61.5% ± 32.3%) (Fig. 6). This suggests that the most abundant ASVs are shared between the crop and the cloacal microbiomes. We further explored the association of richness of these shared ASVs and their relative abundances with the gut length of hosts. We did not find a significant association between host body mass (a proxy for gut length^10^) and the number of shared ASVs between the two regions (lm: R^2^ = 0.0312, F = 0.4835, *p* = 0.4975), indicating that body size did not influence ASV sharing between the two regions. We also did not find a significant association between the combined relative abundance (the proportion of bacterial sequences) of the shared ASVs and body mass (lm: R^2^ = 0.1631, F = 2.921, *p* = 0.1081; Fig. S6). However, the relationship between host body mass and relative abundance of shared ASVs tended to be negatively associated, suggesting that larger birds (with longer digestive tracts) share fewer bacterial sequences between the crop and the cloaca.

## Discussion

We investigated the influence of naturally consumed diets on the crop and the cloacal microbiomes of multiple tropical wild bird species. Aligning with previous studies^10,18^, we found a strong effect of host taxon (i.e., bird order) on the composition of both microbiomes, except for crop microbiomes in dataset1 potentially due to the low sample size. There was also a significant effect of literature-based dietary guilds on cloacal microbiomes (secondary to host taxa)^10,18^, but microbiome similarity did not align with the consumed diet similarity (Fig. 3, Table 2). We did, however, find that the most abundant bacterial ASVs were shared between the crop and the cloaca in bird individuals (Fig. 6), suggesting a little compartmentalisation of gut microbiomes of the bird species included in this study.

Natural diets of bird species analysed herein differ notably from literature-based dietary guilds and showed marked variation between individuals of the same species (Fig. 2), aligning with previous findings from Papua New Guinea^22^. This was expected, as the literature-based dietary guilds represent the average consumption of dietary items of a species that naturally varies with seasonal and regional fluctuations in diet availability^32–34^. The two methods we used to identify dietary components also yielded somewhat contradicting results^35^, potentially due to their inherent limitations. Visual identification can only allow identification of diet components to higher taxonomic levels (e.g., order), and cannot identify smaller or degraded invertebrate body parts, nor can it distinguish between plant taxa^35^. On the other hand, metabarcoding was suboptimal to identify the order Coleoptera (beetles), potentially due to limitations of extracting DNA from hard-bodied beetles or due to the potential use of suboptimal primers^36^ for New Guinean beetles. Metabarcoding can also lead to PCR biases^37,38^ and recover taxa that have been accidentally consumed along with specific diets (i.e., flower mites on figs) or secondarily consumed by the primary food item (e.g., plant material by an herbivorous insect)^35,38^. Thus, a combination of the two methods, accounting for inherent limitations and including manual filtering of known accidentally-consumed diet items, may provide the best representation of the true diets of wild birds. However, one inherent shortcoming in both methods is the lack of quantification of diet items^39^. Less quantitative sampling methods can be problematic when attempting to utilise diet data in downstream analyses, as in our study, in which relative abundances of given diet items are likely to have a strong impact. This, however, should not influence studies investigating wild animal diets, where the main purpose is to understand the dietary niche of a species.

Literature-based dietary guild assignment significantly associated with cloacal microbial composition, but we did not find a strong association between the naturally consumed diets and microbiome similarity (Table 2). This contradiction might be a result of literature-based dietary guilds providing a rough average of the dietary niche of a species, without accounting for dietary intake variation. Furthermore, taxonomically similar bird species tend to belong to similar dietary guilds (e.g., most pigeons are frugivores and most small passerines are insectivores), leading to strong associations with host taxonomy^23^. For example, the consumed diet of individual P46 (a pigeon) only contained insects, while the species is categorized as a frugivore in the literature (Fig. 2). Nevertheless, the microbiome of this individual was similar to other pigeons (Fig. S1). This indicate that the utilisation of generalized dietary guilds from the literature may not reveal actual effects of diet on gut microbiome composition, but rather represent an artifact of the association between the host taxonomy and dietary guilds. However, the observed pattern can also indicate that the long-term dietary and nutritional niche of the species have a stronger effect on shaping gut microbiomes than short-term changes in dietary intake^18^. Thus, long-term studies on diets and microbiomes of the same wild individuals are needed to decipher the realized effect of consumed diets on wild avian gut microbiomes. Diet manipulation studies have demonstrated that the effect of diet on the variation of gut microbiomes is driven by both compositional and macro-nutritional diet content^4^. We were not able to quantify dietary items in individual birds in this study, thus hindering an exhaustive investigation of the realised proportions of each dietary item and their potential influence on gut microbiomes.

The lack of an association between diet similarity and crop microbiomes suggests little influence of diet-associated microbes on bacterial communities in the bird crops. However, microbiome transfer upwards in trophic networks (prey to predator) has been found in blue tits (*Cyanistes caeruleus*), where the microbiomes of specific diet items, at least to some degree, influence the host gut microbiomes^40^. Thus, to better understand the dietary transmission of bird gut bacteria, it will be necessary to investigate the microbes that enter the hosts with their diet. Moreover, we highlight that a large proportion of the crop samples in our study did not amplify, impeding a comprehensive analysis of these microbiomes and potentially driving the observed lack of an association between diet items and crop microbiomes. We do not know the reason for this, but one possible explanation could be the presence of tartar emetic (the chemical compound used to induce regurgitation) in the crop samples that may inhibit bacterial DNA extractions. However, further investigation is required to validate if tartar emetic negatively affects microbial DNA extraction.

Despite the absence of a strong alignment between diet and microbiome similarity, specific cloacal bacterial genera of different hosts were positively correlated with qualitative proportions of dietary items. These correlations varied by bird orders, suggesting that the consumption of compositionally similar diets does not necessarily have the same effect across bird taxa. This supports the prediction that the effect of diet on host microbiomes is secondary to the host taxon^10^ and suggests that bacterial communities can differ based on the dietary differences of individuals within taxa. Further, this result potentially indicates that functionally similar, yet taxonomically different, gut symbionts are associated with similar diet items taken by taxonomically different hosts^4^. However, we note that our analyses were conducted at the host order-level due to a lack of replicates within species. Thus, a thorough investigation including more species with multiple individuals per species is needed to establish how dietary differences impact microbiomes. Nevertheless, our results emphasize that the combined effects of different diet components on certain microbial symbionts drive the overall influence of diet on wild bird microbiomes.

We note that the observed results may underestimate the effect of natural diets on gut microbiomes, as our diet and cloacal microbiome sampling was conducted simultaneously. Diets that actually influence the composition of the observed cloacal microbiomes could be different from the ones we sampled, as the microbiomes could represent a response to previously consumed diets. However, birds have fast gut retention times (a few hours)^41,42^ and avian gut microbes respond to dietary changes quickly^4^. Furthermore, both tropical and temperate birds tend to visit the same feeding areas repeatedly^43,44^ and are likely to find similar prey items^45^. This argument is supported by the lack of a variation of consumed diets at the order level within short time periods in tropical lowland birds in New Guinea (Fig. S7—based on Sam et al. 2017^22^). However, conducting diet manipulation studies, where captive individuals are fed with natural food items (based on knowledge of diet data from the same species) will enable us to tease apart how natural diets impact microbiomes.

The comparison of the crop and the cloaca of the same individuals revealed that the composition of microbiomes in the two regions can differ and that only a relatively small number of ASVs are shared between the two (Fig. 6). However, these shared ASVs were the most abundant ASVs, indicating a bacterial abundance-driven sharing of microbes between the foregut and the hindgut. Furthermore, the tendency that these shared ASVs represent a smaller proportion of the bacterial sequences in larger birds compared to smaller birds (Fig. S6), highlights the importance of avian gut length on gut microbial communities^9,10^. Spatial compartmentalization of gut microbiomes has been observed in multiple bird species^24,46–48^. However, species with segregated microbiomes belong to orders (Galliformes, Charadriiformes, and Struthioniformes)^24,46–48^ that have large ceca, an appendage in the lower gut that harbours highly diverse microbial communities and that is responsible for fermentation of plant-derived material^49^, that might play a role in compartmentalisation of microbiomes in birds. The species we investigated, on the other hand, included host orders (Columbiformes, Coraciiformes, Passeriformes, and Psittaciformes) that lack or have highly reduced (vestigial) ceca^49^. Previous work on compartmentalisation of gut microbiomes of Great tits—*Parus major* (a bird species with vestigial ceca) showed weak levels of spatial segregation of microbes along the digestive tract^50^, indicating potential high levels of microbe sharing between the proximal and distal ends of the digestive tract. Thus, our results suggest that avian taxa that have reduced ceca may experience increased transfer of abundant microbes from the crop to the cloaca and that the extent of this transfer depends on the length of the digestive tract.

Overall, our results demonstrate that the impact of diet on gut microbiomes of birds depends on the composition of the consumed diet, while the response of specific bacterial taxa to different diet items can vary between bird taxa. This indicates the suboptimal nature of utilizing literature-based dietary guilds that strongly align with the host taxonomy to disentangle the relative importance of diet on microbiomes. Species-level variation in diet consumption and the malleability of gut microbes to different diet components are conceivable sources of the high individual variation observed in wild avian gut microbiomes. A thorough examination of how different diet items influence the gut microbiomes and their functional profiles across multiple populations and bird communities is thus warranted to decipher the diet-mediated long-term associations between wild avian hosts and their gut bacterial symbionts.

## Methods

### Sample collection

Birds were captured using canopy nets spanning 0 m to 30 m above the ground at a lowland rainforest site in Northern Papua New Guinea (Baitabag/Kakoba site: 5.14 S, 145.76 E) during May and July 2019. A cloacal sample was collected from each individual immediately after the capture using a Copan mini Floq swab® and stored in RNAlater®. Birds were fed with 0.8 cm^3^ of 1.0% antimony potassium tartar (tartar emetic) per 100 g of bird body mass and placed in a sterile container until regurgitation, but for a maximum of 10 minutes^22^. Once the bird regurgitated, half of the liquid phase (avoiding food items) of the sample was collected using a swab (by dipping the swab multiple times in the regurgitated sample) and stored in RNAlater® to investigate the crop microbiome, while the other half, including visible food items, was stored in 95% EtOH for diet identification and metabarcoding. All samples were stored at − 20 °C within 12 h. Overall, we captured 155 individuals belonging to 40 species (five orders) (Table S1). However, we were only able to collect regurgitated samples and cloacal swabs from 63 individuals (62 regurgitated samples and 63 cloacal samples). Species were also assigned to dietary guilds based on the literature^22,51^. We confirm that the experiment involving vertebrates in this study was carried out in accordance with all the relevant ARRIVE guidelines. Birds were captured according to the Czech Republic and Australian guidelines (licenses CZ1062 and ABBBS no. 3173) and the experiment was approved by Papua New Guinea government-issued research permit (permit no. 9902077829). Furthermore, the experiment was approved by the University of South Bohemia, Czech Republic, and performed under the experimental protocol 1511-20424/2018-67. Collected samples were exported under a Papua New Guinea government-approved export permit no. 019422.

### DNA extractions and microbiome sequencing

DNA from swab samples (along with 100 μL RNAlater®) were extracted using Qiagen DNeasy® blood and tissue kit (Hilden, Germany) following the manufacturer’s guidelines with an extended 12–14-h incubation period. Initial PCRs were conducted following Bodawatta et al. 2020^50^. DNA from positively amplified samples was sent to the University of Michigan’s Microbiome Core for MiSeq amplicon sequencing of the v4 region of the bacterial 16S rRNA gene with SA511 and SP701 primer pair using an Illumina platform. We included two control DNA extractions to account for the potential introduction of bacterial contaminants during extraction and two negative samples were sequenced to assess contamination during sequencing.

### Identifying prey items and metabarcoding of regurgitated samples

We used a combination of morphological sorting (visual identification) of the regurgitated samples and DNA metabarcoding to identify the diets of individual birds. Visual identification of the samples was conducted under a stereomicroscope and was based on assigning identifiable remains of diet items to broad taxonomical (i.e., order level) categories^22^. Invertebrate body parts could most often be assigned to order, but many regurgitated samples contained only small body fragments that could not be identified to family or genus. The presence of plant remnants (seeds, pieces of fruits, pollen grains) was recorded, but most of them could not be identified more precisely (Table S2). Thus, for plant identifications we exclusively depend on DNA metabarcoding.

DNA from the full homogenised regurgitated diet samples was extracted the same way as the microbiome samples. We amplified two regions targeting invertebrate (COI) and plant (ITS2) components of the diet. We used the primers, mlCOIintF^52^ and Fol−degen−rev^53^, to amplify a 313 bp long fragment of the COI gene. This primer pair has very high taxonomic coverage and resolution and is among the most suitable primers for COI metabarcoding^54^. We used a standard set of primers to amplify the plant ITS2 region^55^. We performed a separate PCR for each marker.

Our DNA metabarcoding strategy followed the recommendations by Taberlet et al. 2019^56^. We performed three independent PCR replicates for each sample and included blanks and PCR negative controls. The primer design incorporated 8 bp long tags in both the forward and reverse primer, which allowed us to tag individual PCR replicates of individual samples by a unique combination of tags on the forward and reverse primers. We did the PCR in strips rather than plates to limit cross-contamination^57^. Each strip contained seven samples and either a blank or a PCR negative control. PCR conditions for COI included an initial period of 3 min at 95 °C, followed by 35 cycles of 30 s at 95 °C, 30 s at 45 °C, and 1 min at 72 °C; followed by a final extension of 10 min at 72 °C. PCR conditions for ITS2 followed Bell et al. 2017^58^. We pooled an equal volume of the PCR product from all samples, separately for COI and ITS2, and purified the resulting amplicon pools using magnetic beads (Agencourt AMPure PCR purification kit). Library preparation was done by a PCR-free protocol with Illumina adaptors added by ligation at SEQme (Czech Republic) and the library was sequenced on Illumina NovaSeq 6000 SP 2 × 250 bp, using 1/10 of the capacity of one sequencing lane.

We processed the sequencing data using OBITools^25^ according to the recommendations for filtering and cleaning the data following De Barba et al.^59^ and Taberlet et al^56^. Prior to taxonomic assignment, we clustered the sequences into OTUs with a 97% similarity threshold using the sumaclust software^60^. We used the central sequence from each cluster to assign taxonomic identity to the OTUs. We used the BOLDigger software^61^ with the “diggerhit” method of taxonomic assignment, which submits the sequences to BOLD (http://www.boldsystems.org/), extracts similar sequences, and infers the most likely taxonomic assignment. Identification at the level of species, genus, family, and order required the similarity between the cluster centre and the reference sequences of at least 0.98, 0.95, 0.90, and 0.85, respectively. Hence, for each marker (COI and ITS2) and each PCR replicate we obtained a list of OTUs with taxonomic assignment and abundance (number of reads). To filter out possible contaminants and sequencing errors, we retained only OTUs detected in all three PCR replicates. Finally, we removed any invertebrate OTUs with a taxonomic identification of known avian symbionts (e.g., feather mites and parasites), as these do not represent actual dietary content of a host (Table S3).

For further analyses, diets were examined at the order level (with few exceptions), as we used data acquired from both visual identification and metabarcoding individually and in combination for downstream analyses. However, when combining the data from the two identification methods, we were only able to combine invertebrate identifications from visual data with the metabarcoding, as we were unable to assign plants into higher hierarchical groups (e.g., orders or families) through visual identification. In order to combine these two methods, we utilised a conservative approach by only determining the presence or absence of orders in each diet sample, as we were unable to gather accurate quantities or volumes of each diet component ^39^. For example, with metabarcoding we acquired different numbers of sequences belonging to each dietary taxon, while with visual identification we only identified different body parts of the taxa.

### Microbiome sequences analyses

MiSeq sequences were analysed using the DADA2 pipeline^27^ within QIIME2^28^. Sequences were categorized to ASVs with 100% similarity. ASVs were then assigned to taxonomy using the Silva 132 bacterial reference database^62^. Mitochondrial, Chloroplast and Archaeal sequences were removed using the QIIME2 pipeline. The two control extractions yielded only three ASVs with low number of sequences (four sequences in control 1 and 291 in control 2). These ASVs were only detected in a few of the experimental samples, and always in very low abundances (Table S10). The lack of consistent presence and the rarity of these ASVs imply that they could not have influenced the patterns we see in crop and cloacal microbiomes. Similarly, the two sequencing negative samples included three ASVs, one of which was found in relatively high abundance (31.8% ± 28.9%) in five experimental samples (Table S10). However, since these samples were placed in different locations of the sequencing plate, the ASV was not omnipresent across experimental samples, and only two sequences appeared in the negative control, contamination during sequencing is unlikely. To not bias analyses of experimental samples, we consequently did not remove this ASV from the dataset.

Samples with less than 1,000 total sequences were removed from subsequent analyses. The majority of the microbiome samples failed during sequencing or quality filtering, yielding only a total of 19 crop microbiomes and 39 cloacal microbiome samples. After quality filtering we divided the dataset into three groups: dataset 1. individuals with both successfully sequenced crop microbiomes and diet data (12 individuals), dataset 2. individuals with both cloacal microbiomes and diet data (27 individuals), and dataset 3. individuals with both successfully sequenced crop and cloacal microbiomes (17 individuals). Statistical analyses of the data were conducted in R 4.0.3^63^ and three datasets were analysed independently. Due to differences in sequencing depth, each dataset was rarefied to even sampling depth using the sample with the lowest number of sequences [dataset 1 (Table S4): 1,226 sequences, dataset 2 (Table S7): 1,406 sequences, and dataset 3 (Table S9): 1,353 sequences,] using the rarefy_even_depth function in phyloseq package^26^ (Fig. S2).

### Statistical analyses

Alpha diversities (Chao 1 richness estimate, Shannon’s diversity index) were calculated using the diversity function in the microbiome package^29^. Statistical differences among different categories were investigated using non-parametric Kruskal–Wallis (KW) tests and pairwise differences were tested using Dunn’s post-hoc tests using the FSA package^64^. Microbial community differences were calculated using Bray–Curtis (weighted) and Jaccard (unweighted) dissimilarity matrixes in the phyloseq package^26^. We investigated the effect of literature-based dietary guilds and host taxonomic order (due to limitations of multiple individuals per species we focused on host taxonomy at order level) using PERMANOVAs with the adonis2 function in the vegan package^30^, with “by” parameter set for “margin” to account for marginal effects of the tested variables. Community-level differences were visualized using non-matric multidimensional scaling (NMDS) and principal coordinate analysis (PCoA) plots in phyloseq package^26^.

First, we investigated whether diverse diets lead to an increase in microbial richness (Chao1) using linear models. Then we examined whether crop and cloacal microbiome compositions reflect the diet component similarities (datasets 1 and 2), using Pearson’s correlations between microbiome dissimilarity and diet dissimilarity (individual diet data from visual identification, metabarcoding—both at OTU-level, order-level, and combined) measured with Jaccard dissimilarity index using Mantel tests in the vegan package^30^. The significance of the observed correlations was assessed using 10,000 random permutations. We further explored whether diets influence the microbiomes of bird orders differently by conducting the same analyses individually for each order. To investigate whether individual dietary items impact bacterial genera differently, we examined the correlations (Pearson’s correlations) between relative abundances of bacterial genera and the proportion of particular plant or invertebrate orders in individual diets using the taxa.env.correlation function in the microbiomeSeq package^31^. Here we conducted analyses separately for plant and insect orders and used the combined diets from visual and metabarcoding identifications. We grouped the hosts into orders to decipher whether associations between bacterial genera and diet components differ between bird orders. Significant values were adjusted using Benjamini–Hochberg corrections to reduce the false discovery rate of significant correlations. This was only done for dataset 2 as we did not have enough replicates for each host order in dataset 1.

To investigate similarities between crop and cloacal microbiomes (dataset 3), we utilised PERMANOVA tests^30^. Then to assess the magnitude of the microbiome that is shared between the crop and the cloaca, we investigated both the proportion of shared ASVs and bacterial sequences represented by these ASVs in the two microbiomes. Finally, to explore whether the microbiome sharing between the crop and the cloaca is driven by the gut length of the birds we investigated the association of shared ASVs and their sequence abundances with the bird body mass. Host body mass was utilised as a proxy for the gut length, where larger birds tend to have longer digestive tracts and species-level body masses were acquired from the literature^65^. All illustrations and figures were generated using ggplot2^66^ and viridis packages^67^, and Microsoft PowerPoint.

## Supplementary Information

Supplementary Information 1.

Supplementary Table S1.

Supplementary Table S2.

Supplementary Table S3.

Supplementary Table S4.

Supplementary Table S7.

Supplementary Table S8.

Supplementary Table S9.

## Data Availability

MiSeq amplicon sequencing data (microbiomes) and metabarcoding sequences of diets are submitted to the sequence read archive (SRA) repository at GenBank (MiSeq data: Bio project PRJNA673614, COI sequences of diets: Bio project PRJNA778330, and ITS2 sequences of diets: Bio project PRJNA781139).
