# Supplementary material for: Specific gut bacterial responses to natural diets of tropical birds
Source: Sci Rep. 2022 Jan 13;12:713. doi: 10.1038/s41598-022-04808-9 (PMC8758760; doi:10.1038/s41598-022-04808-9)
Supplement: Supplementary file 1 — Supplementary Information 1. [file 41598_2022_4808_MOESM1_ESM.pdf]

Specific gut bacterial responses to natural diets of tropical birds

Kasun Bodawatta[1*], Irena Klečková[2], Jan Klečka[2], Kateřina Pužejová[2,3], Bonny Koane[4], Michael Poulsen[5], Knud A. Jønsson[1], and Katerina Sam[2,3]

[1] Natural History Museum of Denmark, University of Copenhagen, Copenhagen, Denmark (KHB: bodawatta@snm.ku.dk; KAJ: kajonsson@snm.ku.dk)

[2] Biology Centre of Czech Academy of Sciences, Institute of Entomology, Ceske Budejovice, Branisovska 31, 37005, Czech Republic (IK: irena.slamova@gmail.com, JK: jan.klecka@entu.cas.cz, KP: KatyPuje@seznam.cz, KS: katerina.sam.cz@gmail.com)

[3] Faculty of Science, University of South Bohemia, Ceske Budejovice, Branisovska 1760, 37005, Czech Republic

[4] New Guinea Binatang Research Centre, Madang, Papua New Guinea (BK: bkoane15@gmail.com)

[5] Section for Ecology and Evolution, Department of Biology, University of Copenhagen, Copenhagen, Denmark (MP: mpoulsen@bio.ku.dk)

*Corresponding author

Supplementary Figures

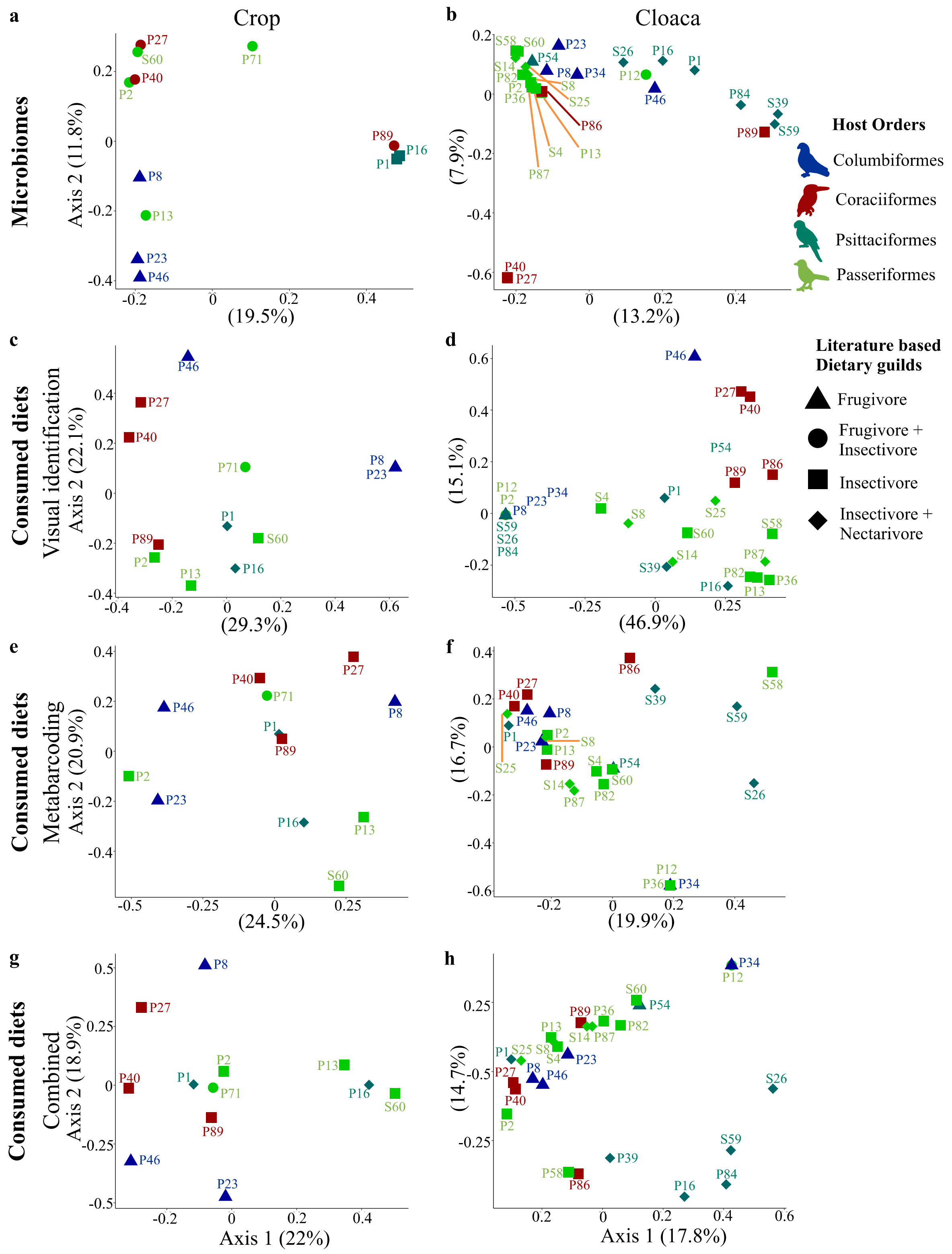

Fig. S1. **a**. Crop (dataset 1) and **b**. Cloacal (dataset 2) microbiome similarity of tropical birds measured with Jaccard dissimilarity distances. Individual IDs are given near points. Colours represent the bird order while shapes represent the literature assigned feeding guilds.

Similarities of consumed diets (Jaccard dissimilarity) of individuals in data set 1(**c, e, g**) and dataset 2 (**d, f, h**). Panels **c** and **d** represent diet similarity based on visual identification, while panels **e** and **f** represent diet similarity based on metabarcoding and panels **g** and **h** represent diet similarity based on combined methods.

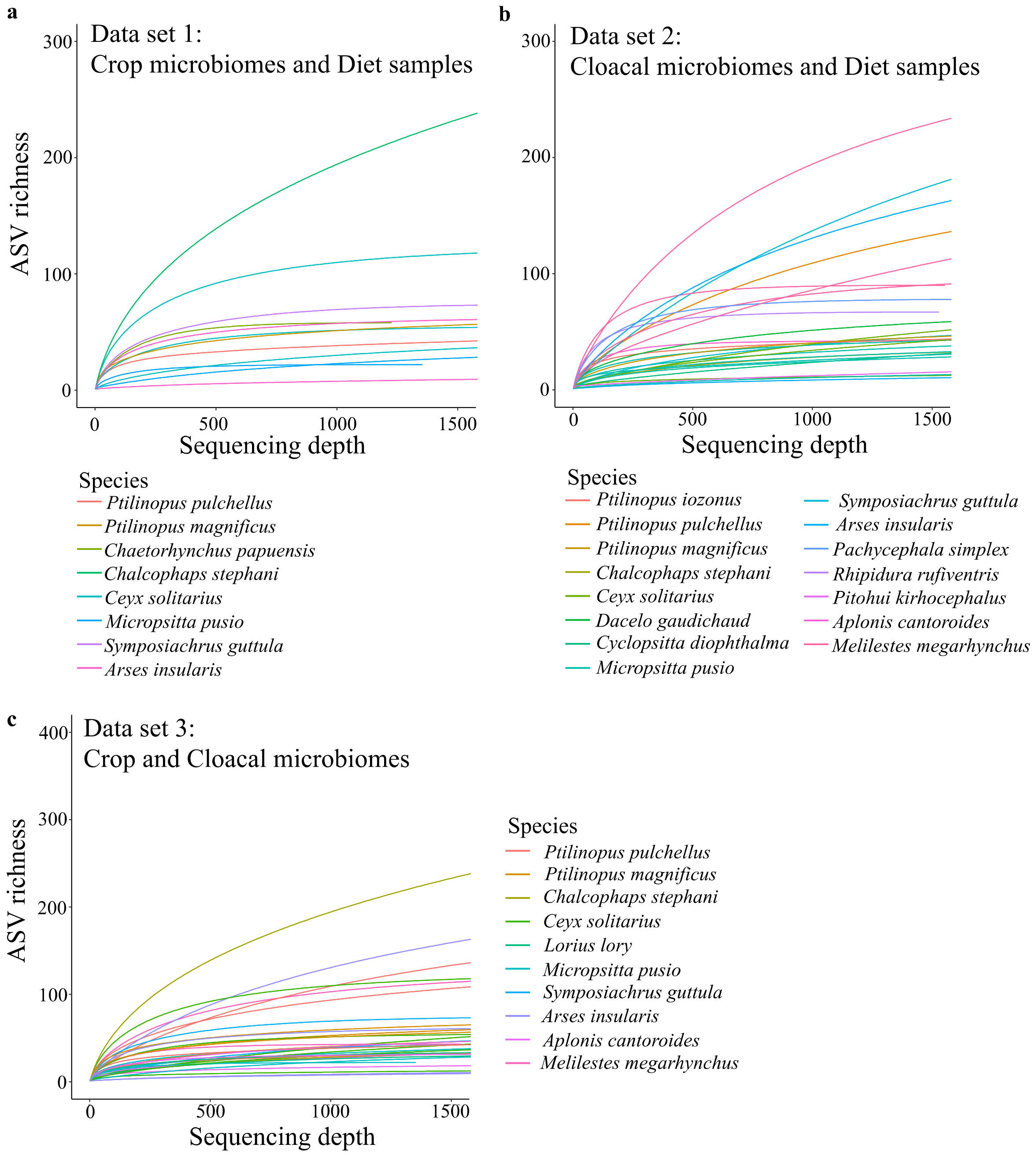

Fig. S2. Rarefaction curves of three data sets: Samples with both crop microbiome and diet samples (**a**: rarefied at 1,226 sequence depth), samples with both cloacal microbiome and diet samples (**b**: rarefied at 1,406 sequence depth) and samples with both crop and cloacal

microbiome samples (**c**: rarefied at 1,353 sequence depth). Lines are coloured according to the species.

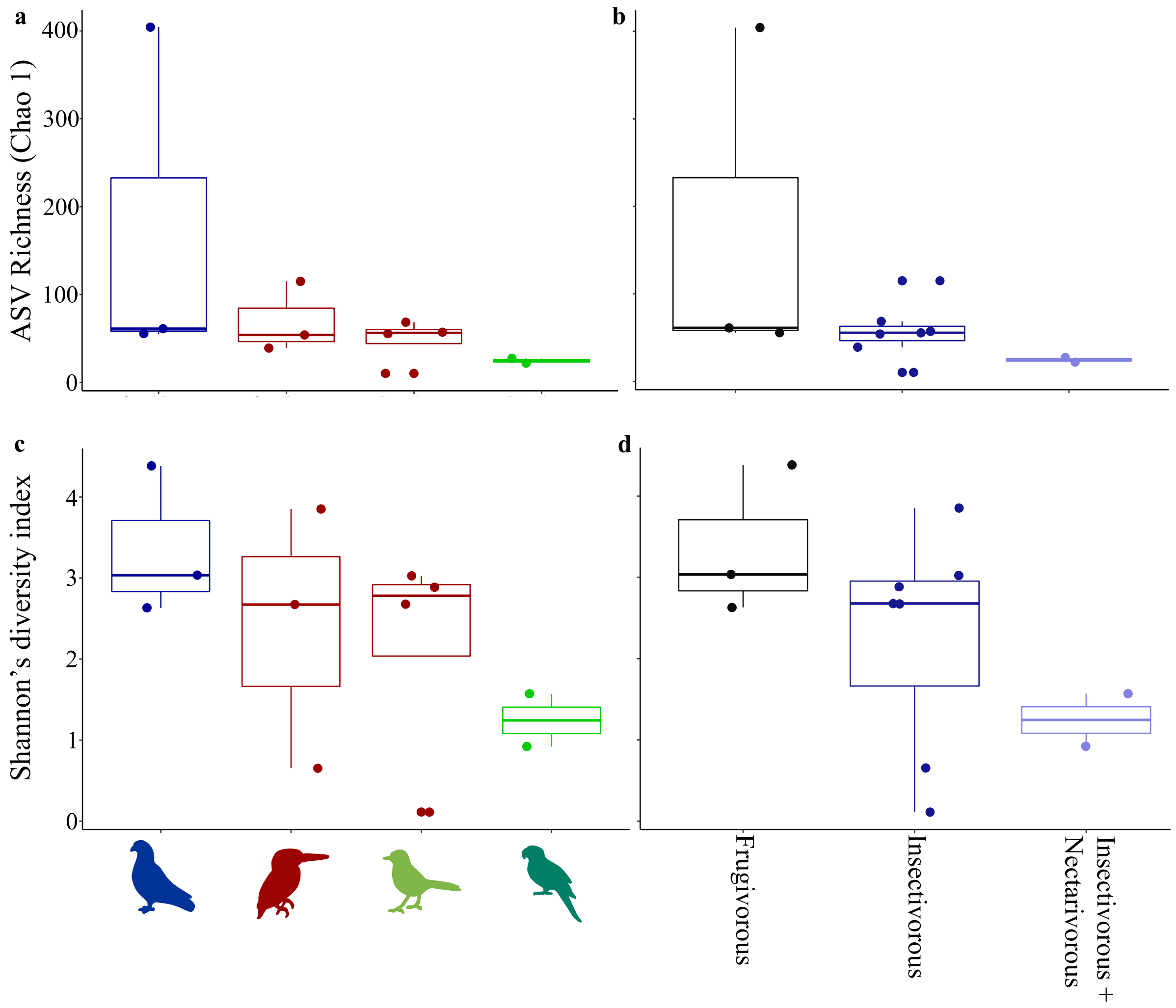

Fig. S3. Box plots depicting crop microbiome ASV richness (Chao1) (a and b) and Shannon's diversity index (c and d) of different bird orders and Literature assigned feeding guilds.

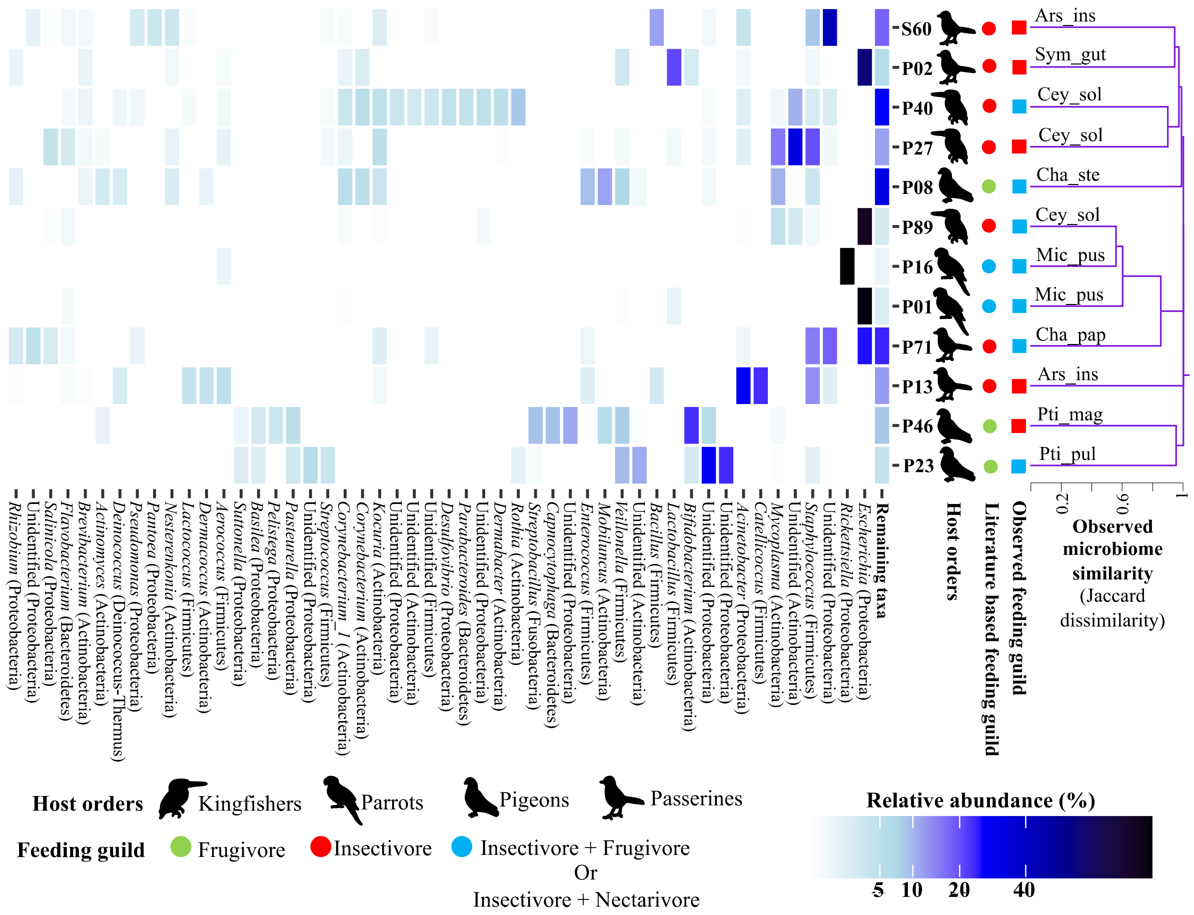

Figure S4. Relative abundance of top 50 bacterial genera in crop microbiomes. Dendrogram represent the microbiome community similarity between individuals based on Jaccard distances. Literature assigned feeding guilds and the observed (based on the combined diet data) feeding guilds of the individuals are indicated next to the bird order icons. Species codes (based on Figure 2) are given near the individual IDs.

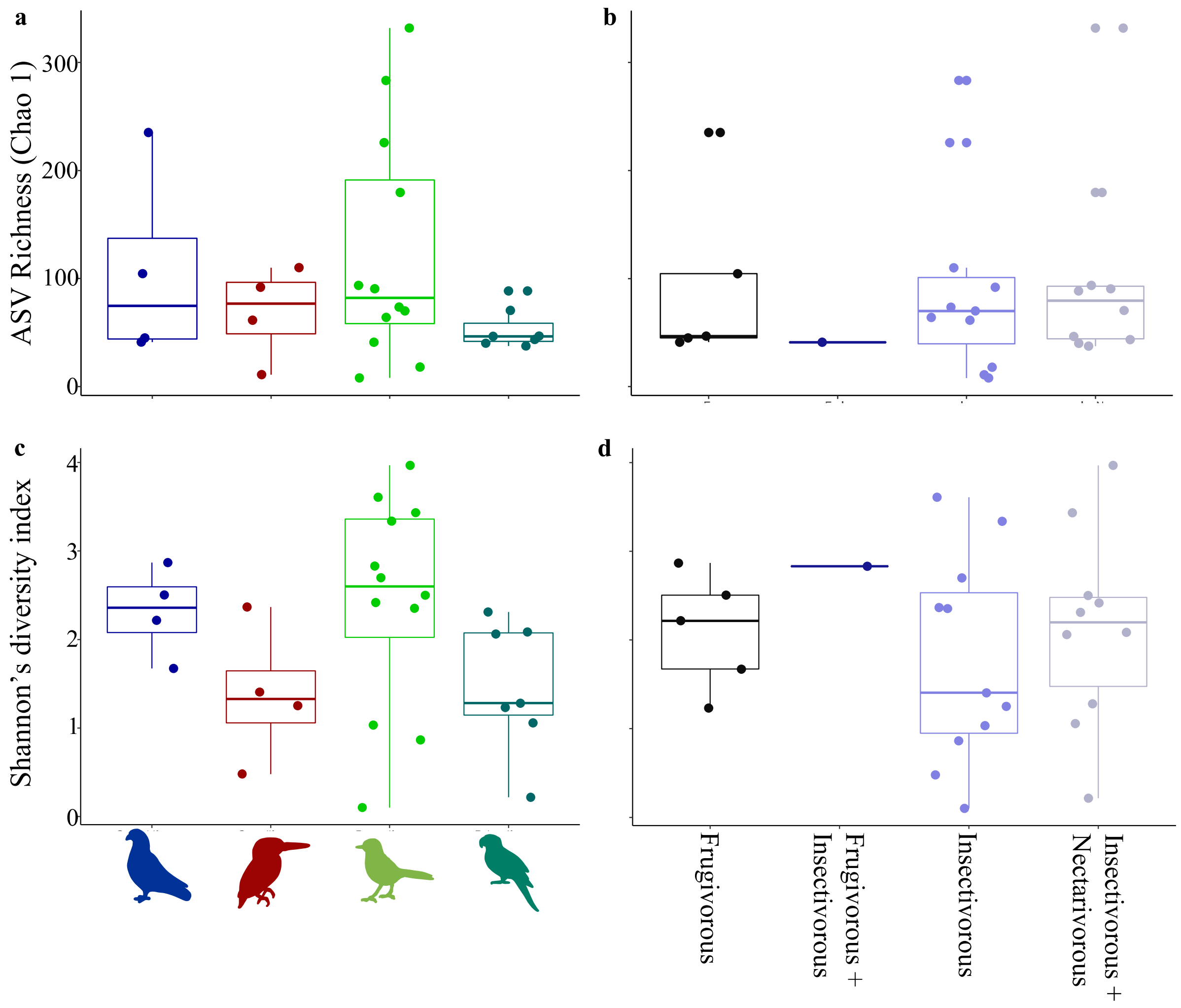

Fig. S5. Box plots depicting cloacal microbiome ASV richness (Chao1) (a and b) and Shannon's diversity index (c and d) of different bird orders and Literature assigned feeding guilds.

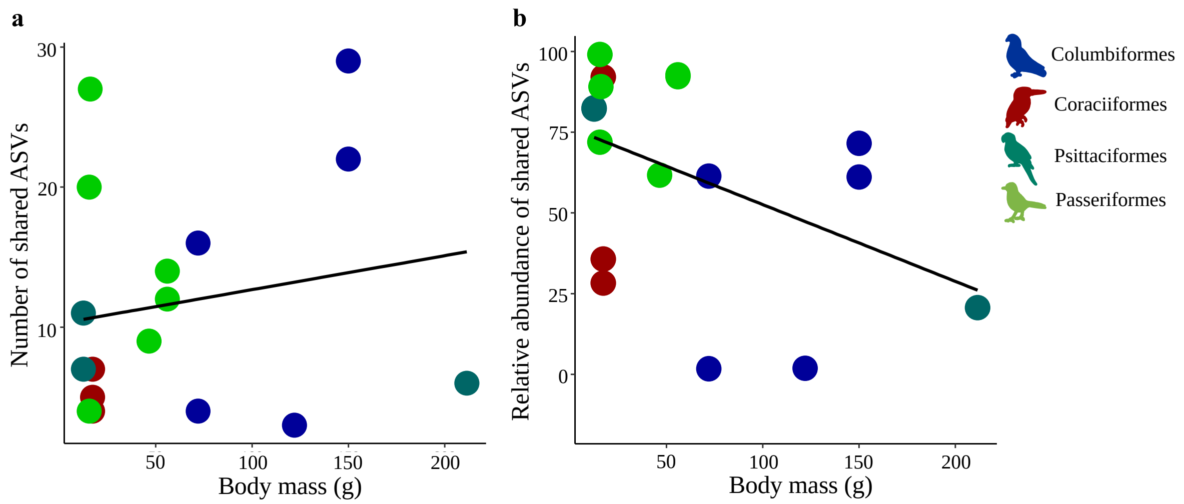

Figure S6. Associations between host body mass and number of shared ASVs (**a**) and proportion of bacterial sequences belonging to these sequences (**b**) in crop and cloacal microbiomes. Grey area represents the standard error of the data.

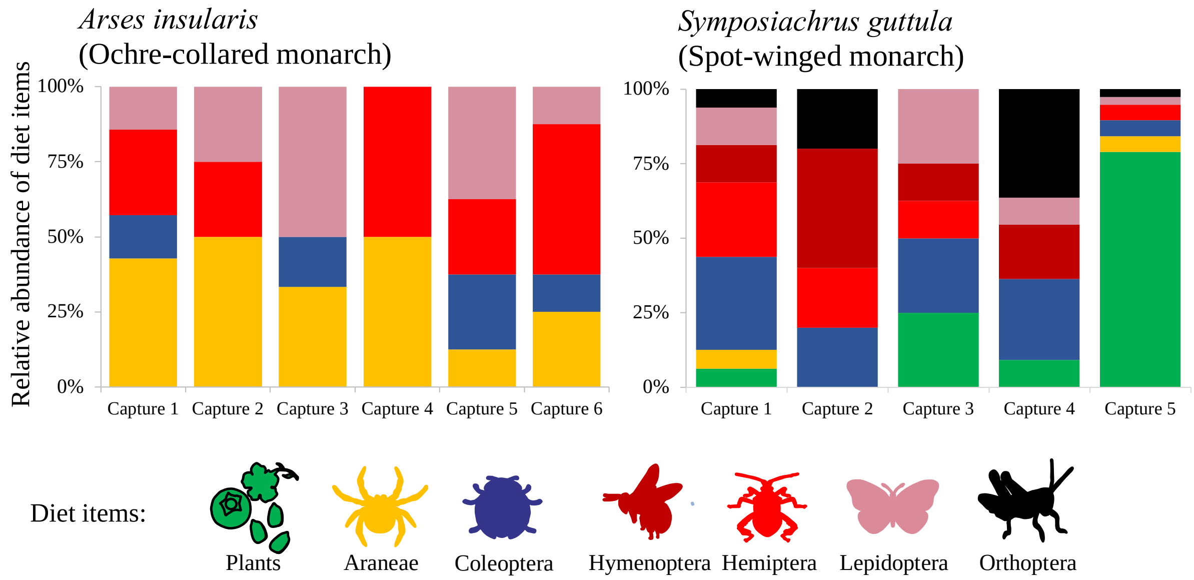

Figure S7. Relative abundance of visually identified diet items from repeated diet collection from the same individual from two bird species (*Arses insularis* and *Symposiachrus guttula*) in Papua New Guinea based on Sam et al. 2017. Individuals were captured during 16 days long survey in Wanang Conservation area, a lowland forest site located ca. 150 km inland from the lowland locality where samples were collected for this study.

Supplementary Tables

Table S1. Total sampled individuals, indicating the successful characterisation of microbiomes and consumed diets (Bold font: Individuals with successful characterisation of diets and cloacal microbiomes, Gray font: individuals with successful identification of diets and crop microbiomes, Underlined: individuals with successfully sequenced crop and cloacal microbiomes) (table is in a separate excel sheet).

Table S2. Diet identification of individuals based on morphological sorting (visual identification). Only invertebrate taxa were identified to order level (table is in a separate excel sheet).

Table S3. Diet identification (OTU table) of individuals based on metabarcoding, using universal primers targeting invertebrates (COI) and plants (ITS2). Metabarcoding sequences can be found in PRJNA778330 (COI gene) and PRJNA781139 (ITS2 gene) Bioprojects in the SRA database (table is in a separate excel sheet).

Table S4. ASV table of the dataset 1 (crop microbiomes and diet samples). "RL" in front of the individual code represent the location (crop) of the microbiome. GenBank accession numbers (PRJNA673614) of the samples are given in respective columns (table is in a separate excel sheet).

Table S5. Statistical results of linear regressions investigating the associations between alfa diversities of microbiomes and consumed diets (combined dataset of results from visual and metabarcoding identification).

**Dataset**	**Richness index**	**F**	**R^2**	**p**
Dataset 1 (Crop microbiomes and diets)	Chao 1 richness	0.6179	0.0582	0.4500
	Shannon's diversity	4.336	0.3025	0.0639
	Chao 1 richness	1.383	0.0524	0.2507

Dataset 2 (Cloacal microbiomes and diets)	Shannon's diversity	0.0617	0.0025	0.8058

Table S6. Results of Mantel tests between crop and cloacal microbiome similarities (measured with Bray-Curtis and Jaccard distance matrices) and consumed diet similarities (measured with Jaccard distance matrix) in of individual bird orders. Separate tests were conducted for visual, metabarcoding and combined identification of diets. Bird orders with low number of samples are indicated with N/A.

**Dataset**	**Diet identification**	**Bird Order**	**Microbiome distance matrix**	**Mantel r**	**p**
Dataset 1 (Crop microbiomes and diets)	Visual	Columbiformes	Bray-Curtis	-0.3592	0.6667
			Jaccard	-0.3650	0.6667
		Coraciiformes	Bray-Curtis	0.8011	0.3333
			Jaccard	0.8081	0.3333
		Passeriformes	Bray-Curtis	-0.4614	0.8333
			Jaccard	-0.471	0.8333
		Psittaciformes	Bray-Curtis	N/A	N/A
			Jaccard	N/A	N/A
	Metabarcoding	Columbiformes	Bray-Curtis	N/A	N/A
			Jaccard	N/A	N/A
		Coraciiformes	Bray-Curtis	-0.6623	0.9999

			Jaccard	-0.6535	0.9999
		Passeriformes	Bray-Curtis	N/A	N/A
			Jaccard	N/A	N/A
		Psittaciformes	Bray-Curtis	N/A	N/A
			Jaccard	N/A	N/A
	Combined	Columbiformes	Bray-Curtis	0.9878	0.3333
			Jaccard	0.9888	0.3333
		Coraciiformes	Bray-Curtis	0.9231	0.3333
			Jaccard	0.9276	0.3333
		Passeriformes	Bray-Curtis	-0.3756	0.7916
			Jaccard	-0.3888	0.7917
		Psittaciformes	Bray-Curtis	N/A	N/A
			Jaccard	N/A	N/A
Dataset 2 (Cloacal microbiomes and diets)	Visual	Columbiformes	Bray-Curtis	-0.0242	0.7500
			Jaccard	0.0015	0.7511
		Coraciiformes	Bray-Curtis	0.4977	0.3333
			Jaccard	0.5077	0.3333
		Passeriformes	Bray-Curtis	-0.1958	0.9197

			Jaccard	-0.1909	0.9163
		Psittaciformes	Bray-Curtis	-0.3729	0.9619
			Jaccard	0.0996	0.3345
	Metabarcoding	Columbiformes	Bray-Curtis	N/A	N/A
			Jaccard	-0.0829	0.5000
		Coraciiformes	Bray-Curtis	-0.1931	0.6667
			Jaccard	0.1686	0.2917
		Passeriformes	Bray-Curtis	0.0783	0.2679
			Jaccard	-0.0587	0.6615
		Psittaciformes	Bray-Curtis	0.1114	0.3492
			Jaccard	0.0814	0.3476
	Combined	Columbiformes	Bray-Curtis	-0.0665	0.5001
			Jaccard	-0.0829	0.5000
		Coraciiformes	Bray-Curtis	0.3778	0.2917
			Jaccard	0.3876	0.2917
		Passeriformes	Bray-Curtis	-0.2604	0.9613
			Jaccard	-0.2521	0.9641
		Psittaciformes	Bray-Curtis	0.1495	0.3008

			Jaccard	0.0204	0.4278

Table S7. ASV table of the dataset 2 (cloacal microbiomes and diet samples). “CS” in front of the individual code represents the location (cloaca) of the microbiome. GenBank accession numbers of the samples are given in respective columns (table is in a separate excel sheet).

Table S8. Pearson’s correlations between cloacal bacterial genera, and insect and plant orders (from the combined diet identifications). Significant correlations are indicated with asterisks (*0.05>p<0.001, **0.001>p<0.0001, ***p<0.0001) (table is in a separate excel sheet).

Table S9. ASV table of the dataset 3 (crop and cloacal microbiomes). “RL” in front of the individual code represent the crop microbiomes while “CS” represents cloacal microbiomes. Gen Bank accession numbers of the samples are given in respective columns (table is in a separate excel sheet).

Table S10. ASVs found in the DNA and negative control samples and their relative abundance in the whole dataset, along with the number of experimental samples and the relative abundance (average ± standard deviation) of these ASVs in the microbiome samples.

Control DNA extractions/ negatives for PCRs	Lowest-level classification of the ASV	Number of sequences belonging to ASVs found in controls and their relative abundance in the entire dataset are given within parenthesis.	# experimental samples where the ASV was found after quality filtering. (Average relative abundance ± SD of these ASVs represented in experimental samples where we observed the ASV)
DNA extraction Control 1	Dermacoccaceae (Actinobacteria)	2 (0.0001%)	8 (0.5±0.9%)
	Yersiniaceae (Proteobacteria)	2 (0.0001%)	1 (0.05%)
DNA extraction Control 2	Enterobacteriaceae (Proteobacteria)	291 (0.02%)	1 (0.03%)
Sequencing Negative 1	Budviciaceae (Proteobacteria)	36 (0.002%)	9 (0.9±1.4%)
	Methylomonadaceae (Proteobacteria)	2 (0.0001%)	5 (31.8±28.9%)

Sequencing Negative 2	Intrasporangiaceae (Actinobacteriota)	9 (0.0005%)	2 (0.5±0.5%)
